# Conversion of waste biomass to designed and tailored activated chars with valuable properties for adsorption and electrochemical applications

**DOI:** 10.1007/s11356-023-28824-y

**Published:** 2023-08-16

**Authors:** Katarzyna Januszewicz, Paweł Kazimierski, Anita Cymann-Sachajdak, Paulina Hercel, Beata Barczak, Monika Wilamowska-Zawłocka, Dariusz Kardaś, Justyna Łuczak

**Affiliations:** 1 Department of Energy Conversion and Storage, Faculty of Chemistry, Gdansk University of Technology, Narutowicza 11/12, 80-233, Gdansk, Poland; 2 Institute of Fluid Flow Machinery, Polish Academy of Sciences, Fiszera 14, 80-231, Gdansk, Poland; 3 Department of Process Engineering and Chemical Technology, Faculty of Chemistry, Gdansk University of Technology, Narutowicza 11/12, 80–233, Gdansk, Poland

**Keywords:** Wastewater treatment, Organic pollutants, Heavy metals, Activated carbon, Pyrolysis, Physical activation, Adsorption, Energy storage

## Abstract

Waste biomass, a renewable energy source, is inexpensive material that has great potential in sorption and electrochemical application. The selected waste materials (corncobs, coconut shells, walnuts, and pistachio husks) allow to close the production cycle and enable material recycling, which are important aspects in the hierarchy of waste management. The proposed methodology for production and activation of biochars can be used industrially due to highly porous structure, developed surface area, and sorption ability of the obtained activated carbons (AC). A significant increase (from 4 up to more than 10 times) in specific surface area (SSA) is observed for all samples after the CO_2_ activation process (0.5 h at 800 °C) up to 725 m^2^ g^−1^ for corncobs, 534.9 m^2^ g^−1^ for pistachio husks, 523 m^2^ g^−1^ for coconut shells, and 393 m^2^ g^−1^ for walnut husks. The highest value of SSA is achieved for the AC derived from corncobs. This material is evaluated for use as an adsorbent, revealing 99% removal of Rhodamine B (dye/AC ratio of 0.0017) and 69% removal of chromium (dye/AC ratio of 0.0028). Based on the adsorption kinetics analysis, it is demonstrated that the Cr(VI) undergoes physical adsorption, while RhB undergoes chemisorption. In addition, corncob-derived AC exhibits superior electrochemical performance in 6 M KOH compared to the nonactivated biochar. A specific capacitance of 70 F g^−1^ at 5 A g^−1^ is achieved, along with outstanding rate capability (45 F g^−1^ at 50 A g^−1^) and cycling stability (94% at 10 A g^−1^ after 10,000 cycles). In contrast, the nonactivated sample shows only 34 F g^−1^ at 5 A g^−1^ and 13 F g^−1^ at 50 A g^−1^, with a stability of 91.4%.

**Supplementary Information:**

The online version contains supplementary material available at 10.1007/s11356-023-28824-y.

## Introduction

Affordable, readily manageable, and highly scalable energy storage systems are emphasized as a primary remedy for tackling the challenges involved with a limited amount of fossil fuels, thereby enabling the utilization of renewable energy and the establishment of a sustainable energy society. As the reserves of fossil fuels continue to diminish and environmental pollution worsens, the paramount challenge humanity faces today is to transform the current energy landscape and tap into clean and renewable energy sources. To confront these issues, particular attention is directed towards low-cost, easily manageable, and highly scalable energy storage systems, which are recognized as key solutions. The implementation of closed-cycle production in all industries, aimed at developing more sustainable and economic processes, promotes the utilization of waste products in various applications (Bałdowska-Witos 2019). The production process of biomass sorbents serves as a notable example of closed-cycle production. Currently, activated carbons are widespread materials used for gas separation and purification (Calvo-Muñoz et al. 2016), water and wastewater treatment (Jutakridsada et al. 2016), energy storage (Zhang et al. 2020; Zou et al. 2020), catalysis, fuel cells (Pal et al. 2018), and others (Chang et al. 2019). However, the high production cost of activated carbon also encourages the search for more cost-effective production technologies using by-products and biomass waste.

Therefore, during the experimental work, physical activation was used due to the low cost of the activation agent and the generation of fewer waste and disposal problems compared to the chemical method. Currently, extensive research is underway to produce and modify biochar derived from waste biomass for various applications, including the adsorption of pollutants. However, the type of raw material, pyrolysis conditions, activation method, and conditions determine the properties of the resulting material (Marsh and Rodriquez Reinoso 2006). Due to the distinct physicochemical properties of biochar derived from different biomass, the effect and mechanism of adsorption of various pollutants differ considerably. Despite the multitude of studies conducted on this subject, the results are inconsistent. Up until now, the selection of precursors for manufacturing carbon electrode materials primarily relied on fossil-based resources such as phenol, pitch, and coal. However, this approach entails complex synthesis conditions and is associated with drawbacks such as high energy consumption, environmental pollution, and elevated costs (Bagreev et al. 2004; Zhang et al. 2017). By utilizing different types of low-cost biomass as precursors with inherent porous structures, abundant heteroatoms, and natural availability, it is possible to effectively address these aforementioned issues and produce carbonaceous materials. Highly conductive carbon materials, such as activated carbons, are used as electrodes in electric double-layer capacitors (EDLCs). Due to their exquisite power density, EDLCs are considered very attractive energy storage technology available on the market today. One of the most distinctive feature of an EDLC is that its two electrodes consist of porous carbon with a high SSA separated by an electronically insulating separator soaked in an electrolyte. After applying a potential to the electrodes, ions from the electrolyte form an electric double layer in close proximity to the charged electrode surface. This process is ideally non-Faradaic, involving only electrostatic separation of charges takes place with neither mass nor charge transfer at the electrode-electrolyte interface. It is commonly known that the higher the electrochemically active surface area (not always directly corresponding to SSA (Frackowiak et al. 2006), the higher the capacitance values (Fic et al. 2018). However, this trend is limited—in materials of SSA exceeding 2500 m^2^ g^−1^ such as graphene, the pore walls may become too thin, leading to a negative effect on the capacitive properties due to the screening of charge (Barbieri et al. 2005). Therefore, pore size, along with their volume, distribution, and accessibility for ions, plays a crucial role in creating EDLCs on large area, resulting in high-capacity values. Physical activation methods cannot achieve the same level of SSA and channel density as chemical activation processes (Li et al. 2020). However, physical activation is a low-cost and environmentally friendly method, which is a significant advantage over the chemical one, which may generate impurities affecting the electrochemical properties of the EDLCs. Hence, we propose physical activation as a greener and lower-cost method to obtain activated carbon for EDLC electrode material, compared to chemical activation.

The objective of this study is to evaluate the effect of the type of biomass, considering elemental composition, cellulose, hemicellulose, lignin contents, and morphology, as well as the duration of CO_2_ activation, on the properties of the resulting activated carbons. For this purpose, four commonly found agricultural waste biomasses were chosen: corncobs, coconut shells, walnut husks, and pistachio husks for pyrolysis. The selected materials are an example of a variety of wastes with high industrial potential due to the amount of waste generated. Shells and husks were chosen to represent hard coals, while the cob of corn naturally possesses an elaborate three-dimensional structure that has the potential as a promising starting material. A low-cost method for enhancing the specific surface area of biochar was proposed, which, under industrial conditions, could be achieved using flue gas. Studying the kinetics of the sorption of carbonaceous materials is crucial for understanding and optimizing the sorption process. This knowledge enables the improved design and efficiency of sorption systems and facilitates the selection of materials suitable for a wide range of pollutants. The product with the highest surface area was evaluated by its application as the adsorbent for model pollutants such as chromium(VI) and Rhodamine B, as well as an electrode material for energy storage devices.

## Materials and methods

### Preparation of activated carbons and their characterization

The four biomass samples—corncobs, coconut shells, walnut husks, and pistachio husks—were selected as substrates for the preparation of activated carbons. The biomass samples (approx. 100 g) were ground to 2–7 mm pieces and pyrolyzed under a nitrogen atmosphere in a steel reactor placed in a muffle furnace (LIFT3.0 + KXP4 R, Neoterm, Wroclaw, Poland). The pyrolysis program was as follows: heating rate 100 °C min^−1^ and well time 30 min at 800 °C. Biochar samples are shown in Figure S1 in Supplementary Materials (SM). The biochar activation process was performed in a horizontal steel reactor placed in a ceramic DMOWSKI-type furnace (ALGA, Gdansk, Poland) equipped with resistance heaters, thermal insulation, and a control system (Figure S2 in SM). The activation process of biochar was performed at 800 °C (the same as pyrolysis temperature). The samples were treated with the CO_2_ activation agent at a constant flow rate of 10 dm^3^·h^−1^. Two different activation times were evaluated, namely 0.5 and 1 h.

Proximate analysis of the raw and pyrolyzed materials, which involved the determination of ash (PN-EN 15403:2011) and volatile matter (PN-EN 15402:2011), was carried out in the muffle furnace (LIFT3.0 + KXP4 R, Neoterm, Wroclaw, Poland). The chemical composition of the biomass samples before and after pyrolytic decomposition was investigated using the CHNS-O Flash 2000 elemental analyzer (Thermo Scientific, Waltham, USA). The sorption properties of the samples, including the BET surface area and the total pore volume of activated carbons, were analyzed by N_2_ adsorption–desorption isotherms at 77 K using a Micromeritics Gemini V200 Shimadzu (Kyoto, Japan) analyzer for SSA. Nitrogen adsorption–desorption isotherms were measured on a surface area analyzer NOVAtouch™ 2 (Quantachrome Instruments) at 77 K for pore size distributions. Before the measurements, all samples were degassed under vacuum at 300 °C for 3 h. Based on the data obtained, the specific surface areas were calculated using the Brunauer–Emmett–Teller (BET) linear equation in the approximate relative pressure range of 0.01 to 0.05. Scanning electron microscopy (SEM) technique (Phenom™ XL G2 Desktop SEM Thermo Fischer Scientific, Waltham, USA) was used to study structural details and the morphology of the pristine and activated biochars. The infrared spectra of the materials were detected using a Fourier transform infrared spectrometer (FT-IR) (PerkinElmer Frontier, USA) using the KBr pellet method (Merck, CAS: 7758-02-3) pellet method, at a resolution of 4 cm^−1^ in the spectral range from 500 to 4000 cm^−1^.

### Adsorption studies

Adsorption experiments were performed in triplicate, and the results presented here represent the average of the three experiments. The standard deviation was used to calculate measurement uncertainties. The removal efficiency and adsorption capacity of the activated biochar at equilibrium were determined using the following equations (Yin et al. 2019).

$$\%\;rem=\frac{\left({C}_0-{C}_e\right)}{C_0}\cdot\;100$$

$${Q}_e=\frac{\left({C}_0-{C}_e\right)\cdot\; V}{m}$$

where *%rem* represents the adsorption efficiency [%], *Q*_*e*_ represents the adsorption capacity [mg∙g^−1^], *C*_0_ and *C*_*e*_, respectively, represent the initial and equilibrium concentrations of Rhodamine B or chromium(VI) [mg∙L^−1^], *V* – represents solution volume [L], and *m* represents the mass of activated biochar [g].

#### Isotherm study

The sorption properties of the sample derived from corncob, which exhibited the highest specific surface area of 752.8 m^2^∙g^−1^, were investigated using Rhodamine B (RhB) and chromium(VI) as model water contaminants. The efficiency of the adsorption of RhB and Cr(VI) by the activated carbon was evaluated by mixing solutions with varying concentrations (10–50 mg∙L^−1^) with the same quantity of AC, followed by a visual comparison of the solution colors. The impact of AC dosage on Cr(VI) adsorption was examined through adsorption experiments utilizing different amounts of the adsorbent (ranging from 1.0 to 6.0 g∙L^−1^) while maintaining constant parameters such as pH (2.2), initial Cr(VI) concentration (25 mg∙L^−1^), and temperature (20 °C). Subsequently, the solutions after adsorption were filtered, and the degree of discoloration was determined using UV-VIS spectrophotometer at 373 nm (Cr(VI)) and 553 nm (RhB) (UV-VIS Evolution 220; Thermo Scientific, Waltham, MA, USA). The visible color changes observed in the samples, wherein varying amounts of AC were employed, were confirmed by UV-VIS measurement, and the equilibrium concentrations were subsequently calculated.

The acquired experimental data were subjected to nonlinear regression analysis to determine the parameters of the Freundlich, Langmuir, Temkin, and Dubinin-Radushkevich isotherms. Subsequently, an isotherm model that exhibited satisfactory agreement with the adsorption phenomenon was identified.

Parameters that belong to the nonlinear Langmuir isotherm model are determined using the following equation:

$${Q}_e=\frac{C_e\cdot {q}_m}{\frac{1}{K_L}+{C}_e}$$

where *Q*_*e*_ represents the adsorption capacity in the equilibrium time [mg∙g^−1^], *C*_*e*_ represents the adsorbate concentration after adsorption in equilibrium time [mg∙L^−1^], *q*_*m*_ represents the maximum monolayer adsorption capacity [mg∙g^−1^], and *K*_*L*_ is a Langmuir constant related to adsorption capacity [L∙mg^−1^].

The Langmuir isotherm is a model that characterizes the adsorption of a chemical species onto a solid surface as a single monolayer. It assumes that the adsorption sites are uniformly distributed and the adsorption energy is constant for all sites. In this model, the maximum adsorption capacity of a single layer of adsorbate on the surface of the sorbent is represented by the constant q_m_. The ratio of the adsorption rate constant to the desorption rate constant is denoted by K_L_. A K_L_ value less than unity indicates a higher desorption rate constant than the adsorption rate constant, whereas a K_L_ value greater than unity implies the reverse situation (Langmuir 1916).

The nonlinear form of Freundlich isotherm is as follows (Ayawei et al. 2017):

$${Q}_e={K}_F\cdot\;{C}_e^{1/n}$$

where *Q*_*e*_ represents the adsorption capacity [mg∙g^−1^], *C*_*e*_ represents the adsorbate concentration in solution after the equilibrium time [mg∙L^−1^], 1/*n* represents adsorption intensity [-], and *K*_*F*_ is Freundlich constant [L∙mg^−1^].

The Freundlich isotherm is a model that can be used to describe physical adsorption on a heterogeneous surface in situations where the adsorption–desorption equilibrium is roughly 50% of the coverage degree (Wang and Guo 2020). In this model, the Freundlich constant (*K*_*f*_) is defined as the product of the maximum adsorption capacity (*q*_*m*_) and the *A*_0_ constant raised to the power of the separation parameter (1/*n*_*f*_). The constant *n*_*f*_, in turn, is expressed as the quotient of *q*_*m*_ and the product of the gas constant (*R*) and the absolute temperature (T) (Wang and Guo 2020).

There is an increasing use of the Temkin isotherm in the analysis of static adsorption of water pollutants. The following equation illustrates the most common usual form (Chu 2021):

$${Q}_e=B\cdot \ln\;\left({C}_e\cdot A\right)$$

$$B=\frac{R\cdot\;T}{b_T}$$

where *Q*_*e*_ represents the adsorption capacity [mg∙g^−1^], *C*_*e*_ represents the adsorbate concentration at adsorption equilibrium [mg∙L^−1^], *R* represents the gas constant [J∙mol^−1^∙K^−1^], *T* represents the temperature [K], *A*, *B*, and *b*_*T*_ represent the Temkin’s constants.

The Temkin model is a theoretical framework that considers the adsorption of a chemical species onto a solid surface as a multilayer process while disregarding the extremes of high and low adsorbate concentrations (Yang 1993). This model takes into account the impact of indirect interactions between the adsorbent and adsorbate on the adsorption process. Furthermore, it assumes a linear decrease in the heat of adsorption of all molecules within the adsorbate layer with an increase in the degree of surface coverage (Yang 1993).

The nonlinear form of Dubinin–Raduskevich (D-R) isotherm is as follows (Ayawei et al. 2017):

$${Q}_e={Q}_m\cdot\;\exp \left(-{K}_{DR}\cdot\;{\varepsilon}^2\right)$$

$$\varepsilon =R\cdot T\cdot\;\ln\;\left(1+\frac{1}{C_e}\right)$$

$$E=\frac{1}{\sqrt{2\cdot\;{K}_{DR}}}$$

where *Q*_*e*_ represents the adsorption capacity [mg∙g^−1^], *Q*_*m*_ represents the theoretical monolayer saturation capacity in D-R model [mg∙g^−1^], *K*_*DR*_ represents the Dubinin–Raduskevich constant [mol^2^∙J^−2^], *ε* represents Polanyi potential [-], *E* represents mean energy of sorption [kJ∙mol^−1^], *R* represents mean gas constant [J∙mol^−1^∙K^−1^], and *T* represents the temperature [K].

The D-R model is an additional theoretical framework utilized in the investigation of adsorption phenomena, which is predicated upon the principles of Polanyi’s theory. Per Polanyi’s theory, an adsorbent system comprises an adsorption space in which molecules release potential energy. The maximal potential energy is obtained within the pores of the adsorbent material since potential energies remain unaffected by temperature and increase within the surrounding spaces of the adsorbate (Wang and Guo 2020). This isotherm is applicable solely to adsorbate concentrations within a moderate range. Application of the D-R model enables the computation of the average adsorption energy (E), where E values below 8 kJ·mol^−1^ may suggest physical adsorption, while E values between 8 and 16 kJ·mol^−1^ may indicate chemical adsorption processes (Chabani et al. 2006).

#### Kinetic study of adsorption

After a time in the adsorption process, the amount of Cr(VI) and RhB adsorbed by AC will not change due to the achievement of dynamic equilibrium. In case to determine the equilibrium time, the 15 mg of AC (corncob) was added into a 25 mg∙L^−1^ solution of Cr(VI) at pH 2.2 and 50 mg∙L^−1^ solution of RhB at pH 5.70 in 5 mL vials. The solutions were shaken at 20 °C for 2, 5, 10, 15, 30, 45, 60, 90, 120, 180, and 240 min. After filtration, the solution was analyzed using UV-Vis spectroscopy.

To investigate the kinetics of RhB and Cr(VI) adsorption by activated biochar, the fit of the experimental data was analyzed, and the parameters of three kinetic models were determined: pseudo-first-order and pseudo-second-order.

The nonlinear form of pseudo first-order model can be given as follows (Qi et al. 2016):

$${\textrm{Q}}_{\textrm{t}}={\textrm{Q}}_{\textrm{e}}\left(1-\exp\ \left(-{\textrm{k}}_1\cdot \textrm{t}\right)\right)$$

where *Q*_*t*_ and *Q*_*e*_ [mg∙g^−1^] represent the adsorption capacities at time *t* [min] and at equilibrium, respectively, and *k*_1_ is the pseudo-first-order rate constant [min^−1^].

The nonlinear form of pseudo-second-order can be expressed as (Zhang et al. 2018):

$${\textrm{Q}}_{\textrm{t}}=\frac{{\textrm{Q}}_{\textrm{e}}^2\cdot\;{\textrm{k}}_2\cdot\;\textrm{t}}{1+{\textrm{Q}}_{\textrm{e}}\cdot {\textrm{k}}_2\cdot\;\textrm{t}}$$

where *Q*_*t*_ and *Q*_*e*_ [mg∙g^−1^] represent the adsorption capacities at time *t* [min] and at equilibrium, respectively, and *k*_2_ is the pseudo-second-order rate constant [mg∙g^−1^∙min^−1^].

The Weber and Morris model of intramolecular diffusion, on the other hand, is used to describe the diffusion mechanism. According to this model, the adsorption capacity (*Q*_*t*_) varies proportionally to the square root of the process time. The Weber–Morris model can be expressed as follows (Aljeboree et al. 2017):

$${Q}_t={k}_{WM}\cdot\sqrt{t}+I$$

where *Q*_*t*_ means the adsorption capacity [mg∙g^−1^] at time *t* [min], *I* means free expression, and *k*_*WM*_ represents the intramolecular diffusion rate constant [mg∙g^−1^∙min^−0,5^].

### Electrochemical measurements

Electrochemical tests were performed in a three-electrode cell configuration in 6 M KOH aqueous electrolyte. Pt wire coated with a thin layer of active material (activated carbon) served as a working electrode, Ag|AgCl|3 M KCl and Pt mesh served as reference and counter electrode, respectively. The working electrodes were prepared by dip-coating method. The procedure was as follows: active material (activated carbon), conductive additive (carbon black Super C65, Imerys) and binder (poly(vinylidene fluoride) PVDF, SOLEF 5130) in a mass ratio of 80:10:10 were mixed with N-methyl pyrrolidone (NMP) and homogenized for 30 min using ball mill homogenizer (MM200, Retsch, Germany) to obtain a slurry. Pt wire was dip-coated in the slurry, dried at RT and in a dryer at 80 °C for 2 h.

Electrochemical measurements were performed using potentiostat-galvanostat SP-200 (BioLogic, Seyssinet-Pariset, France) under EC-Lab software. Electrodes were characterized using cyclic voltammetry (CV) at scan rates of 5–500 mV s^−1^, galvanostatic charge-discharge with potential limitation (GCPL) at current densities of 5–50 A g^−1^.

The specific capacity of the samples was calculated based on cyclic voltammetry (Eq. (1)) and charge/discharge measurements (Eq. (2)).

1 $${C}_s=\frac{I}{\nu \cdot\;m}$$

where *I* is the current [A], 𝜈 is the scan rate [V s^−1^], and *m* is the mass of the working electrode [g].

2 $${C}_s=\frac{I\cdot\;t}{\Delta E\cdot\;m}$$

where *I* is the charge/discharge current [A], *t* is the charge/discharge time [s], ∆*E* is the potential range [V], and *m* is the mass of the working electrode [g].

## Results and discussion

### Preparation, activation, and characteristics of biochars

The mass losses of the investigated biomass samples after pyrolysis and activation processes are presented in Table S1 (in SM). Pyrolysis resulted in the reduction of the solid fraction mass by 77.9–81.4 wt.% (with a mass efficiency of 18.6–22.1 wt.%). Subsequent activation of the biochar with CO_2_ performed for 0.5 h caused further mass loss of 17.4, 16.8, 29.1, and 30.2 wt.% for corncobs, coconut shells, walnut husks, and pistachio husks, respectively. The mass losses detected after 1 h of CO_2_ activation were higher, especially for corncobs (54.0 wt.%) and walnut husks (49.2 wt.%).

Although the mass loss during pyrolysis is comparable for all samples, their behavior during the activation process varies. The corncob char experienced a mass loss of 17.4 wt.% during the first 0.5 h of the activation process and an additional 36.6 wt.% during the subsequent 0.5 h. In contrast, the pistachio husks lost 30.2 wt.% and 11.5 wt.% during the first and the second 0.5 h of activation, respectively. It is noteworthy that both chars have comparable chemical composition (85–86 wt.% of C and 13–14 wt.% of O, as shown in Table S2).

The results of the proximate analysis results for biochars and activated carbons are presented in Table S2. The low content of mineral, inorganic fraction in the tested biochars was confirmed by a low amount of ash in all biomass samples (0.36–0.78 wt.%). The highest volatile matter was detected for pistachio husks (85.6 wt.%), whereas the lowest was recorded for coconut shells (74.7 wt.%). The elemental analysis of the pristine biochar samples was compared with the activated ones in Table S2.

It is known that the activation process removes unreacted residual oils and gases remaining on the carbon surface after pyrolysis and oxidizes the carbon surface, thereby affecting the porosity and composition of the product. Additionally, an excess of activating agent can lead to an increased oxygen content in the carbon sample. This was observed for activated carbons derived from walnut husks and coconut shells after 1 h activation with CO_2_, which contained up to 43.9 and 46.3 wt.% of oxygen, respectively. However, the coconut char before activation already contains 32.7 wt.% of oxygen, and after 0.5 h of CO_2_ treatment, 34%, the 1-h activation increases the oxygen content by 13.6% and 12.3%, respectively. In contrast, the pristine walnut husks char initially contained only 13.7 wt.% of oxygen, indicating that the activation process more than doubled its oxygen content. On the other hand, the lowest increase in oxygen content, during 1 h of the activation process, was noticed for the corncob char (from 12.8 to 20.4 wt.%). Despite the similar chemical compositions of the pistachio and walnut husks biochars (~ 85 wt.% of C and ~ 14 wt.% of O), the resulting activated carbons displayed significantly different compositions (72.8 and 55.6 wt.% of C for pistachio and walnut activated carbons, respectively; 26.2 and 43.9 wt.% of O for pistachio and walnut AC, respectively). These results confirm that the processes occurring during the activation dependent on the microstructure rather than the chemical composition of the pristine biochar. This observations with the analysis of the mass loss data.

**Fig. 1:**
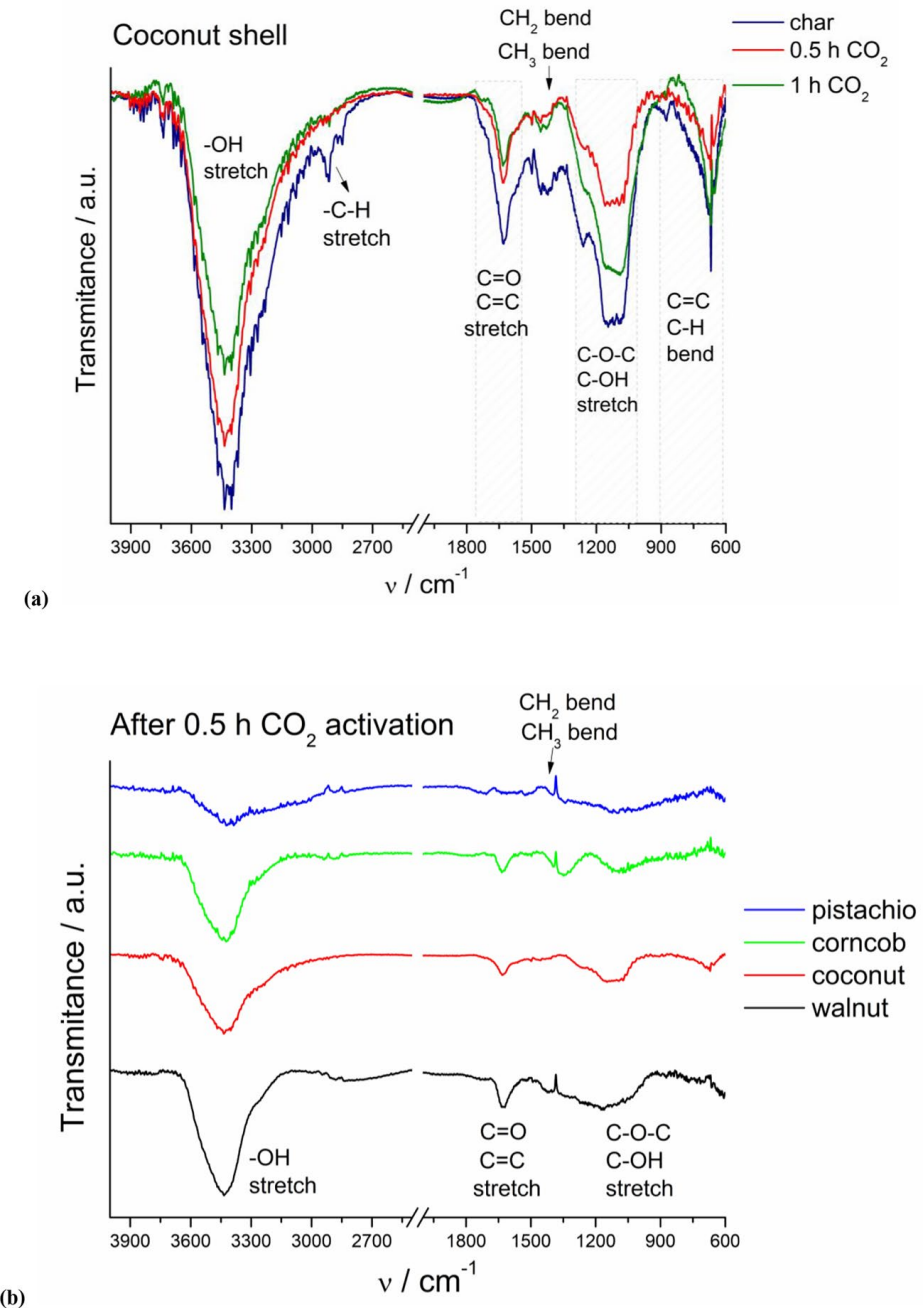
**a** FT-IR spectra of the coconut samples (char, char after 0.5 h and 1 h of CO_2_ activation). **b** Comparison of the transmission spectra of all biomass samples (pistachio husks, corncobs, coconut shells, and walnut husks) obtained after 0.5 h CO_2_ activation

FT-IR spectroscopy was used to monitor changes in the surface composition of the biochars resulting from the CO_2_ treatment. The exemplary FT-IR spectra obtained for the coconut char samples before and after activation are compared in Fig. 1a. The spectrum of coconut char shows a strong, broad band between 3600 and 2800 cm^−1^, which is ascribed to OH stretching vibration of hydroxyl groups. Two bands observed at 2850 cm^−1^ and 2920 cm^−1^ are typical for asymmetric and symmetric stretching of C-H bonds, respectively, present in alkyl groups. The peaks between 1770 and 1550 cm^−1^ may be attributed to carboxyl-carbonate structures, whereas the weak peaks in the range of 1460–1340 cm^−1^ correspond to C-H bending vibration of alkyl groups. The broad bands at 1050–1275 cm^−1^ may be assigned to C-O-C stretching vibrations. The strong bands between 900 and 600 cm^−1^ may be associated with bending vibrations of C=C or C–H bonds. The solid fraction remaining after pyrolysis is rich in the gaseous residues (aromatic compounds, hydrocarbons, and polycyclic aromatic hydrocarbons (PAHs) with alkyl groups such as methyl or methylene) closed in the pores. As shown in Fig. 1a, the intensity of the peaks decreases, and the C-H stretching bands even disappear with increasing surface area of the sample after the activation process. This observation confirms the penetration of the activation agent into the biochar pores resulting in the removal of the gaseous fraction.

The FT-IR spectra of four biomass samples after 0.5 h of CO_2_ treatment are compared in Fig. 1b. The spectra of all biochar samples are shown in Figure S3. The characteristic bands for different functional groups described above are observed with various intensities for all samples. However, for pistachio husks, the intensities of the characteristic bands are much lower than for other samples. This may be due to the differences in the biochar structure and easier diffusion of CO_2_ in the pistachio husks char resulting in enhanced removal of gaseous residues from the pores. This demonstrates the influence of biochar structure on the final properties of the resulting activated carbons.

The results of the surface area analysis performed according to the BET theory for all biochar samples are presented in Table 1. The influence of physical activation with CO_2_ as an activation agent on the biochar samples is pronounced.

**Table 1:** Surface area of the samples before and after the activation process performed at the activation temperature of 800 °C by CO_2_ with a 10 dm^3^ h^−1^ flow rate. *T*_*a*_, activation time; SSA, BET surface area; *V*_*a*_, total pore volume

Biomass	T_a_ [h]	SSA [m^2^ g^−1^]	V_a_ [cm^3^ g^−1^]
Corncobs	0	228	0.14
	0.5	725	0.33
	1	670	0.342
Coconut shells	0	25.3	0.012
	0.5	523.9	0.269
	1	416.0	0.214
Walnut husks	0	32.8	0.017
	0.5	393.0	0.195
	1	380.9	0.201
Pistachio husks	0	10.9	0.005
	0.5	534.9	0.273
	1.0	330.1	0.169

The studied biochars under investigation exhibited SSA ranging from 10.9 to 228 m^2^ g^−1^ prior to the activation process. The highest SSA value among the pristine biochars was found for the corncob sample (228 m^2^ g^−1^). This may be related to the specific cell, thus biochar structure. This sample could be used as an efficient adsorbent directly after the pyrolysis process without activation. The obtained activated carbons exhibit significantly increased specific surface area from 393 m^2^ g^−1^ for walnut husk up to 752 m^2^ g^−1^ for corncob sample. The activation process proved to be most effective for the pistachio husk biochar, as its SSA increased nearly 50-fold (from 10.9 to 534.9 m^2^ g^−1^) after 0.5 h of CO_2_ activation. Conversely, the corncob sample exhibited the smallest increase in SSA (4.27 times), likely due to its initially high SSA as a pristine biochar. However, prolonged exposure to CO_2_ resulted in a lower SSA of the carbon material. The excess of the activator causes severe surface oxidation resulting in a degradation of micropores and mass loss. After an hour of CO_2_ activation, the SSA of the tested samples decreased by 3 to 38% compared to the SSA of the samples after 0.5 h of activation.

The nitrogen adsorption–desorption isotherms recorded for the corncob samples (pristine biochar and activated for 0.5 h), presented in Figure S4(a), represent type I isotherm with H4 hysteresis loop, typical for micro-mesoporous systems. Fast adsorption in low p/p_0_ region is typical for microporous materials. The isotherm loop of the biochar sample is not closed, which suggests the presence of very narrow slit micropores. Furthermore, the pore size distribution for both samples was approximately 1.90 nm, as shown in Figure S4(b-c).

Table S3 presents the summary of the content of biomass components: cellulose, hemicellulose, and lignin, based on literature reports (Pointner et al. 2014; da Silva et al. 2015; Li et al. 2018b; Szyszlak-Bargłowicz et al. 2018). Corncobs and pistachio husks contain almost identical amounts of lignin and cellulose, the difference being only in hemicellulose content, 36 vs 25% for corncobs and pistachio husks, respectively. Both samples assessed have a similar chemical composition and lose the same amount of mass during pyrolysis. However, they behave differently during the activation process, SSA of the corncob sample increases by 3.1 times, while for the pistachio husks, the increase is more than 50 times, but the FT-IR spectra of the activated samples (Fig. 1b) look similar. Coconut shells and walnut husks have almost identical content of cellulose, hemicellulose, and lignin but significantly different oxygen and carbon contents (Table S2–S3). While both samples lose similar mass during pyrolysis, they behave differently during the activation process—walnut husks lose more mass during activation, and their SSA increases 12-fold, whereas the coconut shells lose less mass, but their SSA increases more than 20-fold. Therefore, it is difficult to draw firm conclusions based on the content of cellulose, hemicellulose, and lignin. On the other hand, Silva Lacerda et al. (da Silva et al. 2015) showed that a higher amount of native cellulose in the AC precursor leads to increase SSA and improved adsorption properties.

The surface morphology of biochars produced from different precursors varies significantly (see Figure S5(a-d)). The biochar from corncob, the sample with the highest SSA, has an orderly network of channels (several to tens of μm in diameter) with wall widths of about 2–3 microns. Each wall has a multitude of smaller, circular holes with diameters on the order of hundreds of nanometers. The structure of corncob biochar is regular and looks like interconnected perforated tubes (Figure S5(a)). Similarly, coconut char exhibits a regular structure but is more compact with tightly packed tubes (Figure S5(b)). The tubes are polygonal in cross-section and have rib-like walls. On the other hand, walnut and pistachio husks have a more irregular, sponge-like structure after the carbonization process (Figure S5(c) and (d), respectively). The difference is that the biochar from walnut has larger pores than biochar from pistachio husks.

Energy dispersive X-ray (EDX) mapping analysis of all four biochars showed surface carbon content of more than 60% (see Figure S5(a), (b), (c) and (d) for, corncobs, coconut shells, walnut husks, and pistachio husks, respectively). Note that the surface composition always differs from the bulk composition. However, the EDX mapping confirms the presence of two main components carbon, and oxygen, with no other elements on the surface of the carbonized materials.

Significant differences can be seen in the SEM micrographs after the activation process for 0.5 h and 1 h compared to the carbonization process (Figure S6). CO_2_ flowing through the samples at elevated temperature unblocks already existing pores and eliminates adsorbed contaminants. The removal of undesirable contaminants is visible in the SEM images of pistachio husks-derived activated carbon (Figure S6 D2 and D3) in comparison to the pyrolyzed material (Figure S6 D1). The gaseous CO_2_ acts as the oxidizing agent and reacts with carbon in the sample. Consequently, carbon monoxide is formed and subsequently removed from the furnace with the forced gas flow. Therefore, the already existing pores are enlarged, and new ones are formed. The development of porosity is evident in the SEM images of the activated carbons and further confirmed by BET measurements across all types of the activated carbons.

Over an extended duration, the gasification process of the solid carbon leads to the merging of adjacent pores, resulting in a reduction in SSA. In this investigation, all the analyzed biochar samples exhibited lower BET surface areas after 1 h CO_2_ treatment compared to the results obtained after 0.5 h CO_2_ activation, with the most significant differences observed for the pistachio husks sample.

The AC sample with the highest specific surface area (corncobs-derived AC after 0.5 h CO_2_ activation, SSA of 752.8 m^2^ g^−1^) was selected for further exploration to demonstrate its potential applications as an adsorbent or as an electrode material for energy storage devices.

### Adsorption assays and its results

#### Effect of activated carbon dosage

**Fig. 2:**
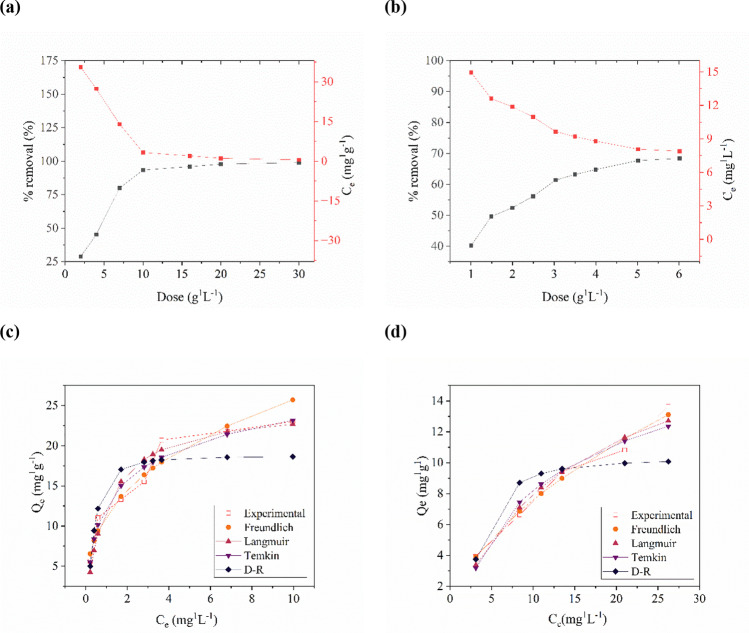
The sorption properties of corncob-derived AC (0.5 h CO_2_ treatment) determined by UV-VIS analysis at 25 °C. AC dosage in the range of **a** 5–150 mg per 50 ppm of Rhodamine B in solution (10 mL), **b** 10–60 mg per 25 ppm of chromium(VI) in solution (5 mL); Freundlich, Langmuir, Temkin, and Dubinin–Radushkevich isotherm constants adsorption of **c** RhB and **d** Cr(VI)

The influence of AC dosage on the adsorption of Cr(VI) was investigated through a series of adsorption experiments employing varying amounts of the adsorbent (ranging from 1.0 to 6.0 g∙L^−1^) while maintaining constant parameters including pH (2.2), initial Cr(VI) concentration (25 mg∙L^−1^), and temperature (20 °C). As depicted in Fig. 2a, b, the gradual increase in adsorbent mass (1.0–3.0 g∙L^−1^) corresponded to a gradual enhancement in the removal efficiency of the metal ion from solutions, ranging from 40 to 62%. Conversely, employing a higher adsorbent dosage (3.5-6.0 g∙L^-1^) resulted in the removal of chromium with an efficiency of 65–70%. This observed increase in chromium removal efficiency with an elevated AC dosage can be attributed to an augmented availability of active sites for adsorbate uptake. Notably, no significant change in adsorption efficiency was observed when the AC dosage was increased from 3.5 to 4.5 g∙L^−1^ and from 5.0 to 6.0 g∙L^−1^, leading to the conclusion that the optimum adsorbent dose was determined to be 3.5 g∙L^−1^. In contrast to the efficiency, the adsorption capacity displayed a decrease with an increasing AC dosage. The highest adsorption capacity, reaching 10.0 mg∙g^−1^, was observed at an AC dosage of 1.0 g∙L^−1^, while a dosage of 3.5 g∙L^−^1 resulted in a capacity of 4.5 mg∙g^−1^.

#### Effect of contaminant dosage

To assess the impact of initial concentrations of Rhodamine B (10–50 mg∙L^−1^) and chromium(VI) (10–50 mg∙L^−1^) on the efficacy of biochar adsorption, experiments were conducted while maintaining constant process parameters such as pH, temperature, and adsorption time. The results of these sorption experiments are presented in Fig. 2. The percentage of dye removal increased with the addition of activated carbon, attributed to the augmented SSA and availability of adsorption sites. However, the efficiency of chromium removal declined from 69 to 41%, while the removal of Rhodamine B decreased from 97 to 80%. The initial concentrations of both chromium(VI) and Rhodamine B exhibited a direct correlation with the adsorption capacity, which measured 13.7 mg∙g^−1^ (50 mg∙L^−1^ Cr(VI)) and 23.0 mg∙g^−1^ (50 mg∙L^−1^ RhB), respectively.

The subsequent phase of the investigation involved determining the adsorption isotherms of the selected contaminants in aqueous solutions. To achieve this, graphs depicting the relationship between adsorption capacity and concentration after adsorption were generated (Fig. 2c, d). The observed shapes of the experimental isotherms suggest the attainment of adsorption equilibrium. Subsequently, the acquired experimental data were subjected to analysis to elucidate the kinetics of the adsorption process.

The parameters of the Freundlich isotherm model were determined based on the obtained data, utilizing the nonlinear form of the equation. The resulting parameter values are presented in Table 2. Notably, the value of 1/*n* was found to be less than unity in both cases, indicating the prevalence of physical adsorption rather than chemical adsorption. Furthermore, a correlation coefficient was computed to evaluate the degree of agreement between the model and the experimental data. The correlation coefficients for RhB and Cr(VI) adsorption were determined to be 0.94 and 0.98, respectively. These high correlation coefficients were further supported by the near-zero values of the chi-square function, affirming the adequacy of the Freundlich model in describing the adsorption behavior of the studied contaminants.

**Table 2:** Model parameters of adsorption isotherms

Type of isotherm	Parameters	RhB	Cr(VI)
Freundlich	*K*_*F*_ [L∙mg^−1^]	11.86±0.66	1.94±0.20
	1/*n* [-]	0.31±0.04	0.58±0.11
	*R*^2^	0.9445	0.9817
	*χ*^2^	1.26	0.12
Langmuir	*Q*_*m*_ [mg∙g^−1^]	23.81±1.49	21.75±2.31
	*K*_*L*_ [L∙mg^−1^]	1.14±0.28	0.054±0.011
	*R*^2^	0.9310	0.9710
	*χ*^2^	16.86	0.30
Temkin	*q*_*T*_ [mg∙g^−1^]	4.60±0.32	0.62±0.12
	*K*_*T*_ [L∙mg^−1^]	15.44±4.21	4.43±0.40
	*R*^2^	0.9605	0.9462
	*χ*^2^	0.75	0.54
Dubinin–Radushkevich (D-R)	*Q*_*m*_ [mg∙g^−1^]	18.72	10.26
	*E* [kJ∙mol^−1^]	2.62	0.48
	*R*^2^	0.8004	0.7211
	*χ*^2^	3.37	1.88

The Langmuir model parameters were also determined using a nonlinear form of the isotherm equation. The parameter *Q*_*m*_, which signifies the adsorption capacity of a single layer of adsorbate, was calculated to be 23.81 mg∙g^−1^ for RhB and 21.75 mg∙g^−1^ for Cr(VI), thereby demonstrating the high potential of the activated carbon produced in the removal of both organic dyes and metal ions from water. In the context of metal ion adsorption, the Langmuir model exhibited a markedly superior fit to the experimental data compared to other models, as evidenced by the values of the linear correlation coefficient (*R*^2^) and the nonlinear correlation *χ*^2^.

An alternative model employed for describing adsorption phenomena is the Temkin model, which accounts for the variation in the heat of adsorption during the process, resulting from the interaction of adsorbent and adsorbate. By utilizing a nonlinearized form of the model, the equilibrium binding constant (*A*) represents the maximum binding energy, and the B constant determines the product *b*_*T*_ (associated with the heat of adsorption) (Piccin et al. 2011; Inyinbor et al. 2016). The obtained *B* values for RhB and Cr(VI) adsorption were approximately 4.5 mg·g^−1^, while the Temkin isotherm constant *A* was determined as 15.4 L·mg^−1^ and 0.6 L·mg^−1^ for RhB and Cr(VI) adsorption, respectively. The linear correlation coefficient for RhB adsorption was found to be the highest among the analyzed models, whereas for Cr(VI) adsorption, the Temkin model exhibited a weaker fit compared to the Langmuir and Freundlich isotherms.

The experimental data obtained were further analyzed using the Dubinin–Radushkevich isotherm model to determine the free energy of sorption (*E*). The linearized form of the isotherm was applied to determine the parameter *Q*_*m*_, representing the maximum adsorption capacity. For the RhB adsorption process, *Q*_*m*_ was determined as 18.72 mg·g^−1^, while for chromium(VI), *Q*_*m*_ was found to be 10.26 mg·g^−1^. These values were considerably lower compared to those obtained with the previously discussed Langmuir adsorption model. The correlation coefficient indicated a weaker fit of the model data to the experimental data. Although the determined adsorption energies in both cases were below 8 kJ·mol^−1^, which may indicate the physical nature of the process, further investigation into adsorption thermodynamics is required to confirm this hypothesis.

Regarding chromium(VI) adsorption, the Freundlich isotherm model exhibited the best fit, as indicated by the higher linear regression coefficient value (*R*^2^ = 0.98) compared to the other isotherms. This finding confirms the formation of a multilayer adsorbate on the surface of the activated carbon and suggests the heterogeneity of the adsorbent. A heterogeneity parameter below unity (1/*n* = 0.56) indicates that adsorption occurs primarily in the low concentration range, and as the initial concentration increases, the rate of increase in adsorbed substance tends to decrease. In a study by (Li et al. 2018a) employing corncob activated carbon, a similar adsorption capacity value of 9.10 mg·g^−1^ was obtained, with a heterogeneity parameter of 0.58 (Li et al. 2018a). Similarly, Zhang et al. (2018) investigated the adsorption of Cr(VI) on activated carbon derived from corncobs and observed a better consistency of the experimental data with the Freundlich isotherm model (*R*^2^ = 0.99) (Zhang et al. 2018). In the present study, the *K*_*F*_ constant was 3.22, and the heterogeneity parameter was 0.39 (Zhang et al. 2018). Another study by Barczak et al. (2022) focused on Rhodamine B adsorption onto activated carbon obtained from corn cobs, examining solely the effect of adsorbate dosage on adsorption capacity, which was determined as 46.6 mg·g^−1^ (Barczak et al. 2022).

#### Adsorption kinetics

**Fig. 3:**
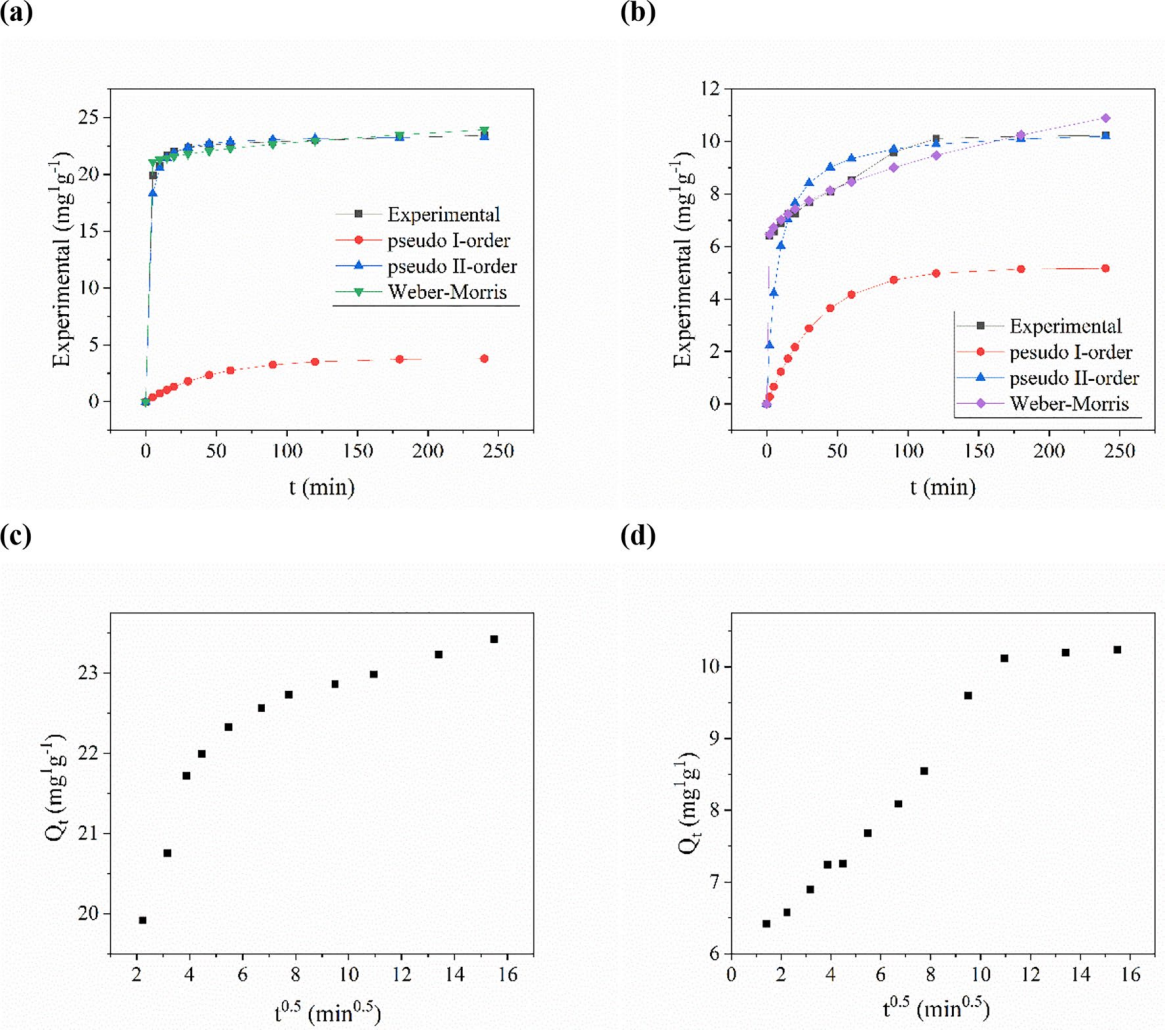
Kinetical graphic of the adsorption of **a** RhB and **b** Cr(VI) made with corn cobs activated carbon. Weber-Morris kinetic plots of **c** Rhodamine B adsorption and **d** chromium(VI) adsorption

Figure 3a and b present the kinetics of RhB and Cr(VI) adsorption, showcasing the time-dependent behavior of the process. It was observed that for chromium(VI), the adsorption efficiency reached 40% after 5 min, whereas for RhB, it was approximately 70%. The efficiency of the adsorption process steadily increased with time, reaching equilibrium after 120 min, where no significant changes in concentration were observed over time. The adsorption capacity also exhibited a gradual increase with increasing processing time, reaching the maximum values at equilibrium at 10.2 mg∙g^−1^ for Cr (VI) and 23.4 mg∙g^−1^ for RhB. These equilibrium values were considered as the experimental adsorption capacities (Qexp) for further calculations.

To further assess the kinetic parameters of Cr(VI) and RhB adsorption onto activated carbon (AC), the pseudo-first-order, pseudo-second-order, and Weber–Morris kinetic equations were employed. These equations were used to analyze the rate at which the adsorption process occurred and determine the corresponding kinetic parameters.

The investigation of adsorption kinetics plays a pivotal role in understanding the mechanism and rate of the adsorption process, both of which are fundamental characteristics of adsorbents. In order to assess these aspects, plots illustrating the relationship between adsorption capacity (*Q*_*t*_) and process time (*t*) were constructed, as depicted in Fig. 3a, b. Notably, an initial rapid increase in adsorption capacity was observed for both adsorbates within the first 15 min, indicating a fast adsorption rate. The experimental data obtained from these studies were subsequently utilized to determine the parameters of selected kinetic models, thereby providing valuable insights into the underlying mechanisms of the investigated adsorption processes. A comprehensive overview of the model fitting results is presented in Table 3.

**Table 3:** The parameters of the kinetic models

Type of isotherm	Parameters	RhB	Cr(VI)
Pseudo-first-order	*k*_1_ [min^−1^]	0.29	0.03
	*Q*_*e*_ [mg∙g^−1^]	4.04	5.18
	*R*^2^	0.90	0.98
	*χ*^2^	198.5	41.41
Pseudo-second-order	*k*_2_ [g∙mg^−1^∙min^−1^]	0.027	0.013
	*Q*_*e*_ [mg∙g^−1^]	23.47	10.51
	*R*^2^	~ 1.00	0.99
	*χ*^2^	0.14	3.99
Weber–Morris (diffusion)	*K*_WM_ [mg∙g^−1^∙min^−0,5^]	0.21	0.35
	*I* [mg∙g^−1^]	20.61	6.01
	*R*^2^	0.71	0.94
	*χ*^2^	0.15	0.23

The pseudo-first-order adsorption model assumes that the rate of adsorption is directly proportional to the equilibrium equation and the instantaneous concentration of the adsorbate. By linearizing this model, it is possible to determine the equilibrium adsorption capacity (*Q*_*e*_) and the adsorption rate constant (*k*_1_) (Dzieniszewska and Kyzioł-Komosińska 2018). However, in comparison to the experimental values (23.45 mg∙g^−1^ and 10.24 mg∙g^−1^, respectively), the determined equilibrium adsorption capacities were found to be 4.04 mg∙g^−1^ (RhB) and 5.18 mg∙g^−1^ (Cr(VI)). It should be noted that the pseudo-first-order adsorption model typically exhibits a good fit only during the initial stage of the adsorption process, as observed in the majority of cases reported in the literature, including the processes investigated in this study.

The pseudo-second-order model assumes that the rate-limiting step in the adsorption process is the chemical interaction between the adsorbent and adsorbate molecules. By linearizing the equation, parameters such as the rate constant (*k*_2_) and equilibrium adsorption capacity (*Q*_*e*_) can be determined. The calculated *Q*_*e*_ values were found to be 23.47 mg∙g^−1^ (RhB) and 10.17 mg∙g^−1^ (Cr(VI)). These values exhibit a closer agreement with the experimental data, as further supported by the high correlation coefficients *R*^2^, which approach unity. The enhanced agreement observed when applying the pseudo-second-order model suggests that the adsorption processes of both rhodamine and chromium(VI) are influenced by the abundance of free active centers on the surface of the adsorbent.

$$2 AC+ Cr{(VI)}_{sol}\overset{k_2}{\to }\ {AC}_2{Cr}_{sol id\ phase}$$

$$2 AC+{RhB}_{sol}\overset{k_2}{\to }\ {AC}_2{RhB}_{sol id\ phase}$$

where AC represents an unoccupied sorption site on activated biocarbon and *k*_2_ is the pseudo-second-order rate constant.

The Weber–Morris model was employed as the final model to analyze the kinetics of the adsorption process and determine the rate-limiting step. This model provides insights into the controlling mechanism of the adsorption process. In cases where diffusion inside the pore governs the process, the relationship between the adsorption quantity (*q*) and the square root of time (*t*^0.5^) is expected to be linear and pass through the origin of the coordinate system. Conversely, a nonlinear relationship suggests that diffusion in the boundary layer is the rate-limiting step (Belhachemi et al. 2009). The determined values of the boundary layer thickness, 20.61 mg∙g^−1^ for RhB and 6.01 mg∙g^−1^ for Cr(VI), indicate that the influence of the boundary layer on the kinetics of the adsorption process is relatively small. The obtained *q* = *f*(*q*^0.5^) relationships, as depicted in Fig. 3c, d, demonstrate a nonlinearity, indicating the presence of three distinct stages in the adsorption process. The first stage corresponds to the instantaneous adsorption on the external surface, followed by intramolecular diffusion within the pores, which limits the rate of the process. The final stage is characterized by a flattened curve, representing the attainment of equilibrium and a significant reduction in the rate of intramolecular diffusion.

For comparison, Ollo et al. (2021) reported the adsorption capacity of Rhodamine B on corn cobs activated biochar to be 0.89 mg∙g^−1^, with a specific surface area of 613 m^2^∙g^−1^. Their findings were in accordance with the pseudo-second-order model (*R*^2^ = 0.99) (Ollo et al. 2021). In another study, Tang et al. (2016) investigated the adsorption of Cr(VI) on active biocarbon derived from corn cobs and observed an equilibrium adsorption capacity of 14.49 mg∙g^−1^ (Tang et al. 2016). Both in our research and the studies cited above, the pseudo-second-order model provided better agreement with the experimental data. This suggests that the adsorbate molecule occupies two active centers (Zhang et al. 2018).

The differences observed in the adsorption capacity (*Q*) between the experimental results obtained in our study, and the literature data can be primarily attributed to the larger specific surface area (925 m^2^∙g^−1^) and variations in the pore size distribution achieved through chemical activation. The higher specific surface area indicates a more microporous structure, which plays a crucial role in adsorption. Conversely, larger diameter pores (meso- and macro-pores) mainly facilitate transport functions. Furthermore, the adsorption process is influenced by multiple factors, including the properties of the adsorbent, such as pore distribution and surface chemistry (surface functional groups and surface charge), as well as experimental conditions like initial adsorbate concentration, process duration, and pH (de Souza et al. 2022). In the work of Sundarabal et al. (2013), the activated carbons derived from corncobs demonstrated a considerably lower adsorption capacity of 4.97 mg∙g^−1^, in compliance with the pseudo-second-order model(Sundarabal et al. 2013).

It is worth mentioning that the adsorption process does not represent the final stage of contaminant removal; rather, the subsequent step involves the regeneration of the carbon adsorbent or the thermal degradation of the adsorbent along with the pollutant. The recycling of activated carbon after the adsorption process is increasingly gaining attention as a significant research topic (Reza et al. 2020; Zhou et al. 2021). Several methods have been described in the literature for this purpose, including ozonation, electrochemical techniques (e.g., electro-Fenton, electro-peroxone), and dielectric-barrier discharge (DBD) plasma. These methods require further investigation and optimization of their parameters. It is crucial to predict the efficiency and consider the economic aspects when developing regeneration methods for activated carbon.

### Electrochemical investigations

**Fig. 4:**
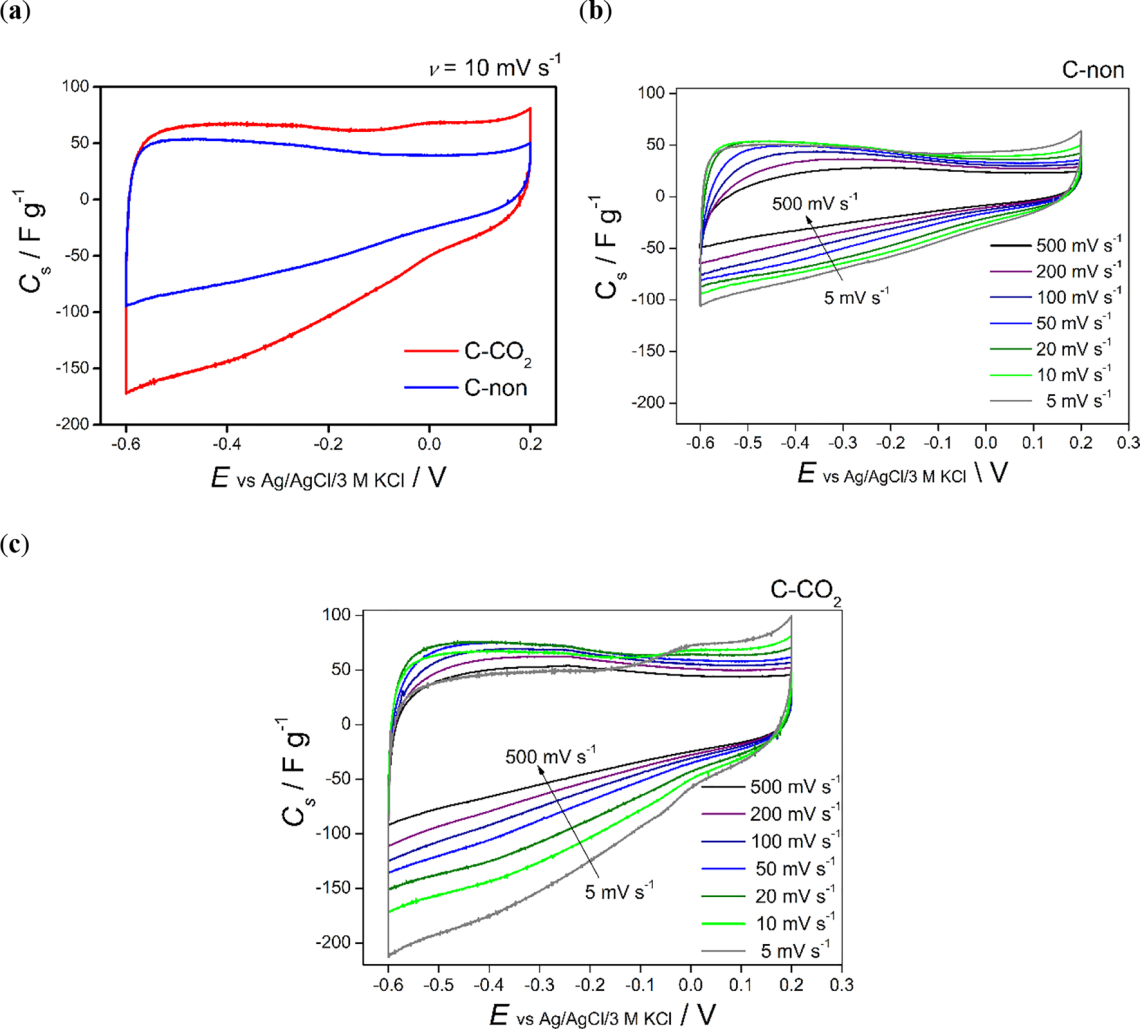
**a** Comparison of CV curves recorded in 6 M KOH at a scan rate (𝜈) of 10 mV s^−1^ for corncobs-derived biochar (C-non) and corncobs-derived activated carbon after 0.5 h CO_2_ treatment (C-CO_2_). **b**, **c** CV curves recorded in 6 M KOH at various scan rates for C-non and C-CO_2_ sample, respectively

The electrochemical properties of the activated carbon derived from corncobs were evaluated through cyclic voltammetry (CV) and galvanostatic charge–discharge cycles. Figure 4a presents a comparison of the CV curves recorded for the corncobs-derived biochar (referred to as C-non) and the activated carbon obtained after 0.5 h of CO_2_ activation (referred to as C-CO_2_).

Both CV curves exhibit a rectangular shape, typical for carbonaceous materials of large specific surface area, showing high capacitance values coming from electric-double layer formation. Oxygen-rich surface functional groups, present in larger amounts in activated samples as confirmed by FT-IR analysis, may also contribute to the capacitance values. The higher specific surface area of the activated sample leads to a significantly higher capacitance value (38.1 F g^−1^ for the biochar sample compared to 64.7 F g^−1^ for the CO_2_-activated sample), proving that the porosity developed during the physical activation process is accessible for the adsorption of ions from the electrolyte.

**Fig. 5:**
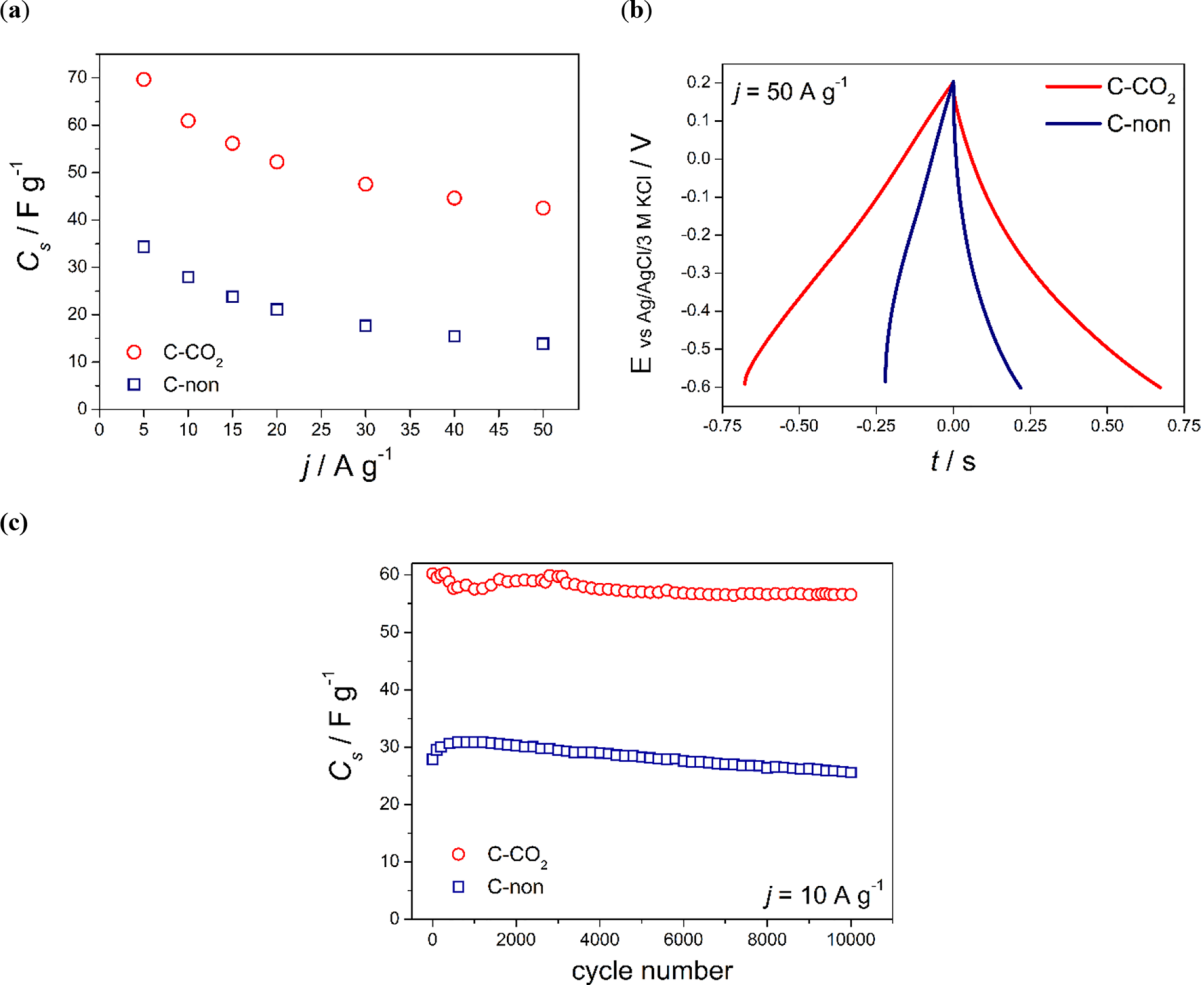
**a** Comparison of specific capacitance (*C*_s_) values calculated from the galvanostatic discharge curves recorded at various current densities for corncobs-derived biochar (C-non) and AC (C-CO_2_). **b** Corresponding charge-discharge curves recorded at high current density (50 A g^−1^) for C-non and C-CO_2_ samples. **c** Electrochemical performance of C-non and C-CO_2_ samples during 10 000 charge–discharge cycles at polarization current of 10 A g^−1^

Figure 5b and c illustrate the CV curves obtained for the biochar and CO_2_-activated sample, respectively, at various scan rates. The area of CV curve corresponding to the capacitance value decreases with increasing scan rate, which suggests diffusion limitation across the electrode layer and possible kinetic limitation of the redox reaction of surface functional groups. However, even at 500 mV s^−1^ the CV shape of the CO_2_-activated sample remains rectangular, and the specific capacitance value reaches 36.4 F g^−1^.

Galvanostatic charge–discharge tests were also performed at various current densities for the corncobs-derived raw biochar and CO_2_-activated carbon. The results are presented in Fig. 5a and b and Figure S5 (a) and (b). Specific capacitance values were calculated from galvanostatic charge–discharge curves using Eq. (2).

The specific capacitance values of the CO_2_-activated sample are twice as high as those obtained for the raw biochar (Fig. 5a), which is consistent with the results obtained from cyclic voltammetry measurements. Charge/discharge curves exhibit a triangular and symmetric shape in the potential range of − 0.6 to 0.2 V (vs Ag/AgCl/3M KCl) with no potential drop even at the high current rate (50 A g^−1^) as presented in Fig. 5b. The charge capacitance values are equal to the discharge ones for both tested samples (Figure S5 (a) and (b) in SM), which prove that highly reversible energy storage processes occur at the electrode materials.

To evaluate the stability of the electrode materials, a prolonged GCPL was performed. The specific capacitance values over 10,000 charge–discharge cycles are presented in Fig. 5c. Both electrodes (CO_2_-activated carbon and raw biochar) exhibit a slight decrease upon cycling (approximately 94% of initial capacitance values after 10,000 cycles). The small fluctuation in capacitance values may come from the temperature changes in the laboratory because the cells were not thermostated.

Activated carbons are electrode materials that are widely used in electrochemical capacitors (Frackowiak and Béguin 2001; Béguin et al. 2014; Manickavasakam et al. 2020; Jiang et al. 2020). Capacitance values are proportional to the specific surface area available for ions from the electrolyte. Therefore, the capacitance depends also on the porosity and pore size distribution within the electrode material, which in turn depend on the structure of the carbonaceous material. The carbons derived from biomass investigated here present various structures, which are, to a different extent, affected by the CO_2_ activation process. Biomass type, thermal treatment and activation conditions influence the morphology, structure, porosity, and SSA of the final activated carbons (Zhao et al. 2016; Tripathi et al. 2016; Januszewicz et al. 2020a, 2020b). More sophisticated, multistage, and more expensive methods combined with chemical activation often led to the production of activated carbons of exceptionally high SSA (Wang et al. 2010, 2012; Du et al. 2013). For example, Wang et al. achieved specific a surface area of 2789 m^2^ g^−1^ for AC by the combination of expansion (heating the corncobs sample in the airtight reactor to the pressure of 1 MPa followed by an adiabatic expansion and sudden cooling) and further KOH activation (4:1 KOH to corncobs char ratio, activation at 800 °C for 1 h under N_2_ atmosphere) (Wang et al. 2010). A high specific surface area with proper porosity leads to outstanding capacitance values. For example, corncob with 1722 m^2^ g^−1^ was obtained in a chemical-activation process (4:1 KOH and 4:1 urea) in 800 °C for 2 h and exhibited the capacity of 303.6 F g^−1^ at 5 A g^−1^ in 6 M KOH (Song et al. 2020). The described activation process is multistage and requires a lot of chemicals, and generates many wastes in comparison to a simple physical activation process. The electrochemical results presented here show that corncob-derived CO_2_-activated carbon may be considered a low-cost electrode material for electrochemical capacitor application.

## Conclusions

Waste biomass is a suitable raw material for activated carbon production. Depending on the type of biomass, thereby its composition, and morphology, the obtained products of pyrolysis (chars) have various porosity and surface area. Moreover, the CO_2_ activation process, performed under the same conditions, causes a different degree of surface development for several types of biomasses. SSA after 0.5 h of CO_2_ activation increases from 4.3 times (for corncobs) to 49.1 times (for pistachio husks). These results show that the biomass composition and microstructure of the biochar significantly affect the final AC properties. Among the four investigated biomass types, corncobs revealed the highest potential for use as activated carbon reflected by the largest specific surface area after pyrolysis (228.2 m^2^ g^−1^) and after 0.5 h of CO_2_ activation process (752.75 m^2^ g^−1^). It is worth to mention that physical activation is a practice of producing activated carbons rapidly and is characterized by fewer disposal issues compared with chemical activation.

An interesting effect was observed by comparing the results of the specific surface area of biochars activated for 0.5 and 1 h. The too long activation process decreased the specific surface area of activated carbons, thus worsening the most valuable property in terms of their potential application. The investigation of different activation processes is significant for several reasons. A shorter activation process increases the mass efficiency of the system, decreases CO_2_ demand, and improves the thermal economy of the process.

Corncobs-derived activated carbon may be used as a cheap adsorbent for wastewater treatment because it efficiently removes Rhodamine B and chromium(VI) (model water contaminants) from the solution. Furthermore, this AC is characterized by decent capacitance values (70 F g^−1^ at 5 A g^−1^), outstanding rate capability (45 F g^−1^ at 50 A g^−1^) and good stability after prolonged cycling (94% of initial capacitance value after 1000 charge-discharge cycles). These properties make the corncobs-derived activated carbon a potential candidate for electrodes in supercapacitors.

## Supplementary information

ESM 1 (DOCX 6680 kb)

## Data Availability

All data generated or analyzed during this study are included in this published article (and its supplementary information files).
