# Supplementary material for: Conversion of waste biomass to designed and tailored activated chars with valuable properties for adsorption and electrochemical applications
Source: Environ Sci Pollut Res Int. 2023 Aug 16;30(43):96977–92. doi: 10.1007/s11356-023-28824-y (PMC10495522; doi:10.1007/s11356-023-28824-y)
Supplement: Supplementary file 1 — (DOCX 6680 kb) [file 11356_2023_28824_MOESM1_ESM.docx]

**Supplementary materials**

**Equilibrium and adsorption kinetic study of waste biomass-derived activated chars as valuable products for**

**wastewater treatment and energy storage.**

Katarzyna Januszewicz ^1,*^, Paweł Kazimierski ^2^, Anita Cymann-Sachajdak ^1^, Paulina Hercel ^2^, Beata Barczak^1^, Monika Wilamowska- Zawłocka ^1,^, Dariusz Kardaś ^2^, Justyna Łuczak ^3^

^1^ Department of Energy Conversion and Storage, Faculty of Chemistry, Gdańsk University of Technology, Narutowicza 11/12, 80-233 Gdańsk, Poland;

^2^ Institute of Fluid Flow Machinery, Polish Academy of Sciences, Fiszera 14, 80-231 Gdańsk, Poland;

^3^ Department of Process Engineering and Chemical Technology, Faculty of Chemistry, Gdańsk University of Technology, Narutowicza 11/12, 80–233 Gdańsk, Poland;

*****Corresponding author: [monika.wilamowska@pg.edu.pl](mailto:monika.wilamowska@pg.edu.pl)

| 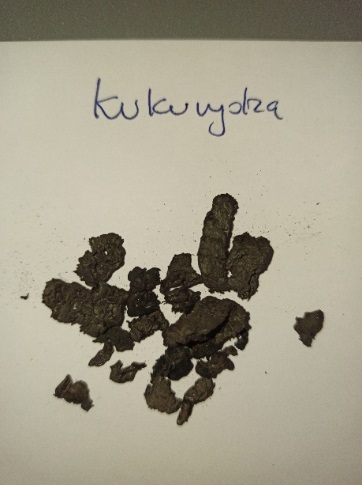1 | 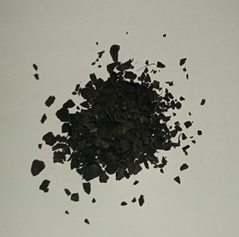2 | 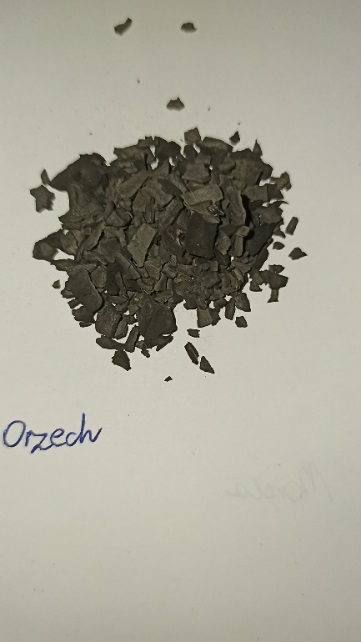3 | 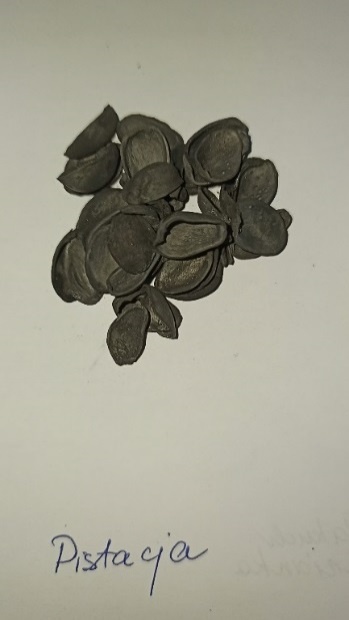4 |
| --- | --- | --- | --- |

**Figure S1.** Biochars derived from pyrolysis of (1) corncobs, (2) coconut shells, (3) walnut husks, and (4) pistachio husks.


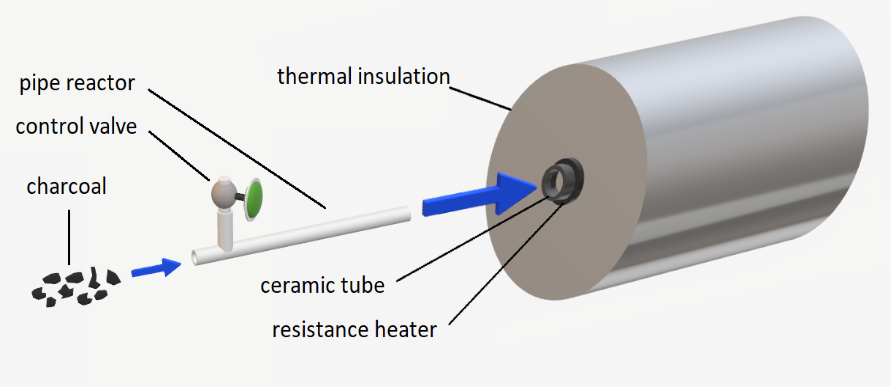


**Figure S2.** Scheme of the lab-scale CO_2_ activation system used in the experiments.

**Table S1.** The mass losses of the samples during pyrolysis and activation processes.

|  | Time [h] | Corncobs | Coconut shells | Walnut husks | Pistachio husks |
| --- | --- | --- | --- | --- | --- |
|  |  | **Mass loss [wt.%]** | | | |
| **Pyrolysis** | 0.5 | 81.4 | 78.5 | 77.9 | 80.2 |
| **CO_2_ activation** | 0.5 | 17.4 | 16.8 | 29.1 | 30.2 |
|  | 1.0 | 54.0 | 20.3 | 49.2 | 41.7 |

**Table S2.** Elemental analysis for the investigated raw materials and obtained activated carbons.

| **Sample** | **Proximate analysis (wt.%)** | | **Elemental analysis (wt.%)** | | | | | | | |
| --- | --- | --- | --- | --- | --- | --- | --- | --- | --- | --- |
|  | **RAW MATERIAL** | |  |  |  |  |  |  |  |  |
|  | **Ash** | **Volatile** | **C** | | **H** | | **N** | | **O** | |
| corncobs | 0.76 | 81.3 | 85.6 | 0.98 | | 0.59 | | 12.8 | |  |
| corncobs, CO_2_ 0.5 h |  |  | 79.1 | 0.56 | | 0.24 | | 20.1 | |  |
| corncobs, CO_2_ 1 h |  |  | 78.7 | 0.61 | | 0.27 | | 20.4 | |  |
| coconut shells | 0.46 | 74.7 | 66.4 | 0.48 | | 0.46 | | 32.7 | |  |
| coconut shells, CO_2_ 0.5 h |  |  | 64.5 | 1.06 | | 0.45 | | 34.0 | |  |
| coconut shells, CO_2_ 1 h |  |  | 53.2 | 0.27 | | 0.24 | | 46.3 | |  |
| walnut husks | 0.48 | 78.3 | 84.7 | 1.31 | | 0.25 | | 13.7 | |  |
| walnut husks, CO_2_ 0.5 h |  |  | 82.4 | 0.53 | | 0.21 | | 16.9 | |  |
| walnut husks, CO_2_ 1 h |  |  | 55.6 | 0.46 | | 0.07 | | 43.9 | |  |
| pistachio husks | 0.36 | 85.6 | 85.0 | 0.61 | | 0.42 | | 14.0 | |  |
| pistachio husks, CO_2_ 0.5 h |  |  | 73.2 | 0.90 | | 0.30 | | 25.6 | |  |
| pistachio husks, CO_2_ 1 h |  |  | 72.8 | 0.48 | | 0.48 | | 26.2 | |  |

**

**

**Figure S3**. FTIR spectra of biochars of the investigated biomass samples.

**(a)**



**(b)
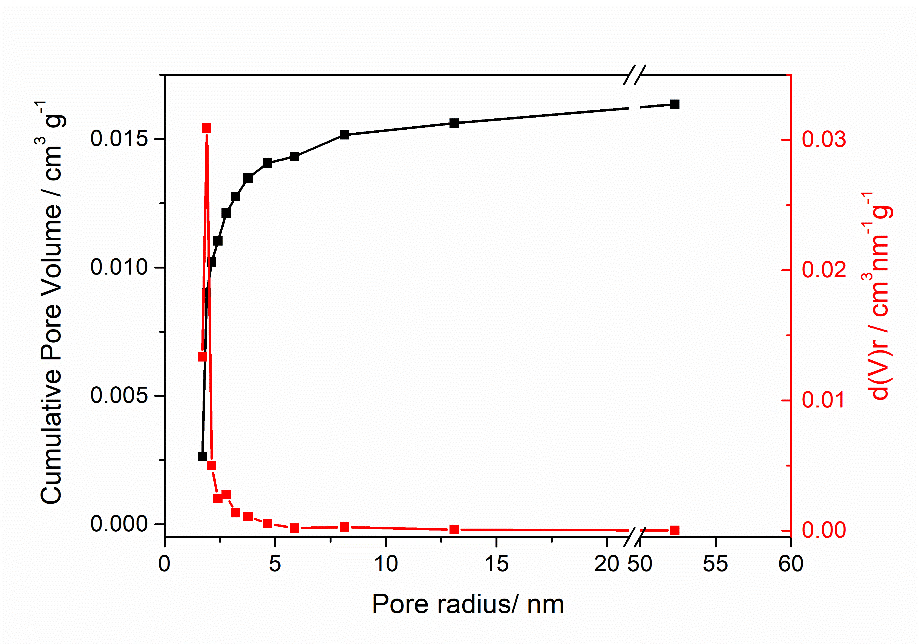
**

**(c)**
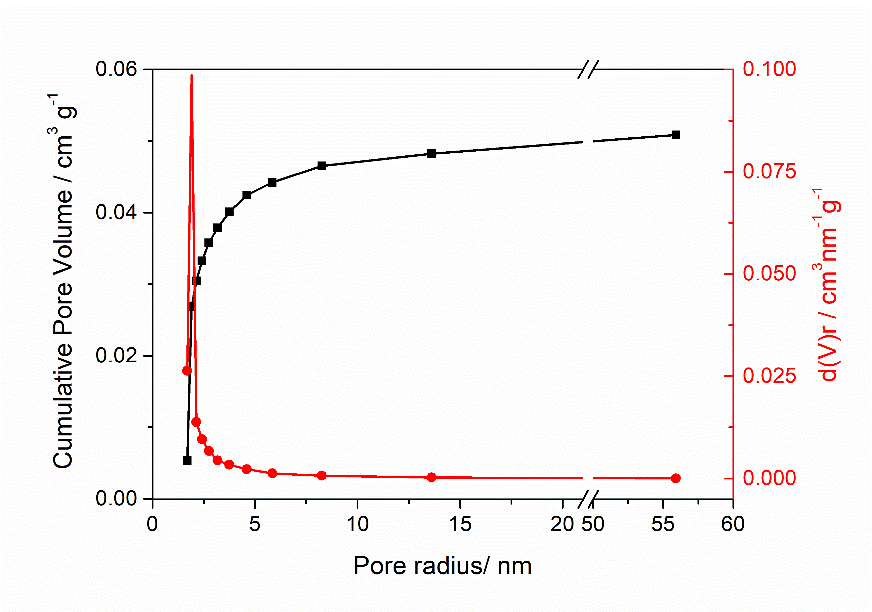


**Figure S4.** Nitrogen adsorption-desorption isotherms recorded for the corncob samples, pristine biochar and activated for 0.5 h (a), pore size distributions of the corncob biochar (b) and activated sample (c).

**Table S3.** Proportion of cellulose, hemicellulose and lignin in the analyzed samples.

| **Biomass** | **Cellulose** | **Hemicellulose** | **Lignin** | | **Ref.** | |
| --- | --- | --- | --- | --- | --- | --- |
|  | **[wt. %]** | | |  | |  |
| corncobs |  |  |  | |  | |
|  | 38.8 | 44.4 | 11.9 | | [1] | |
|  | 27.71 | 38.78 | 9.5 | | [2] | |
|  |  |  |  | |  | |
| coconut shells |  |  |  | |  | |
|  | 34.12 | 22.36 | 28.04 | | [3] | |
|  |  |  |  | |  | |
| walnut husks |  |  |  | |  | |
|  | 32 | 22 | 31 | | [4] | |
|  |  |  |  | |  | |
| pistachio husks |  |  |  | |  | |
|  | 43.08 | 25.30 | 16.33 | | [3] | |
|  |  |  |  | |  | |


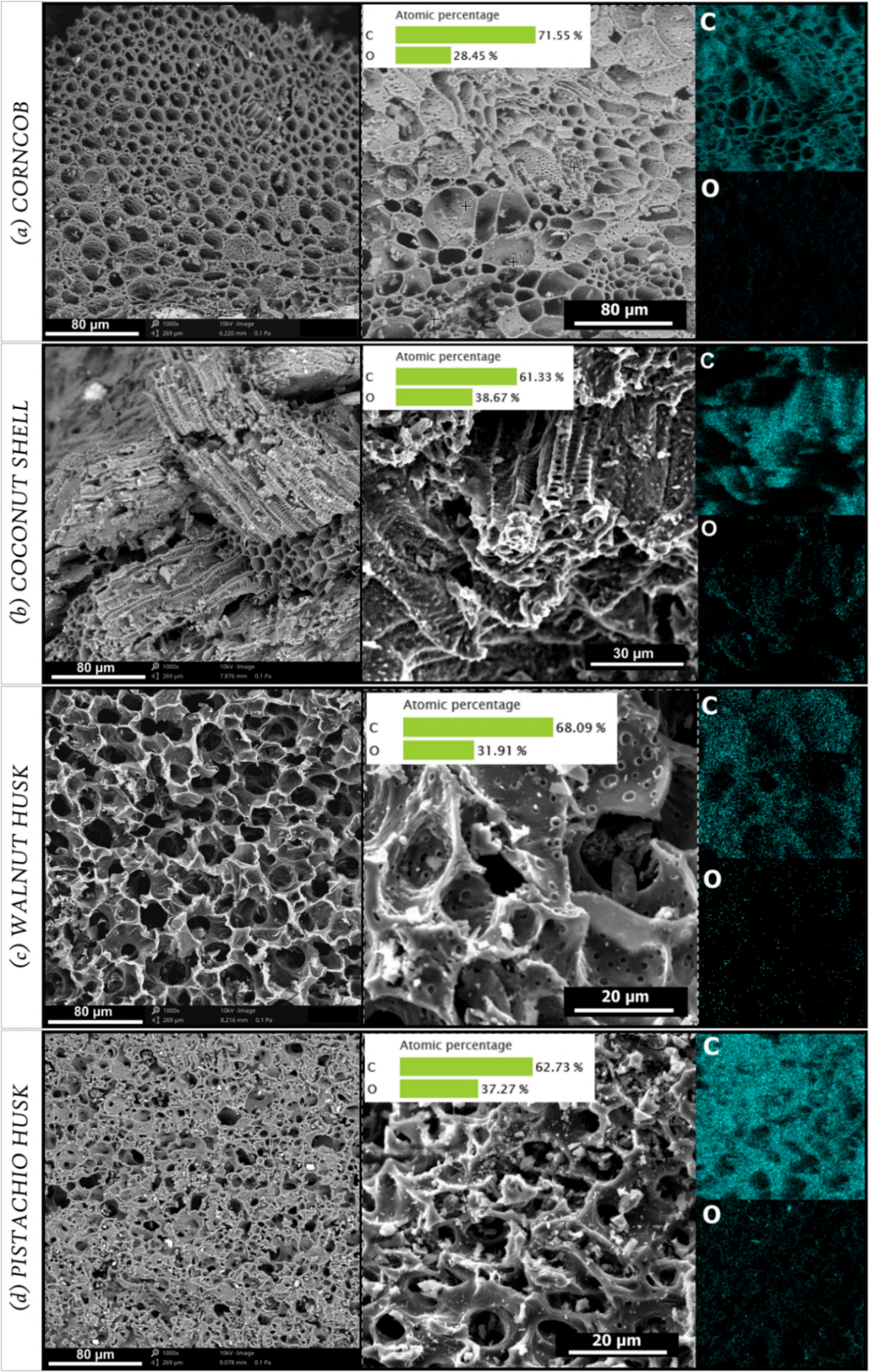


**Figure S5.** Scanning electron microscopy (SEM) pictures and energy dispersive X-ray (EDX) mapping of the investigated biochars: a) corncobs, b) coconut shells, c) walnut husks, and d) pistachio husks.


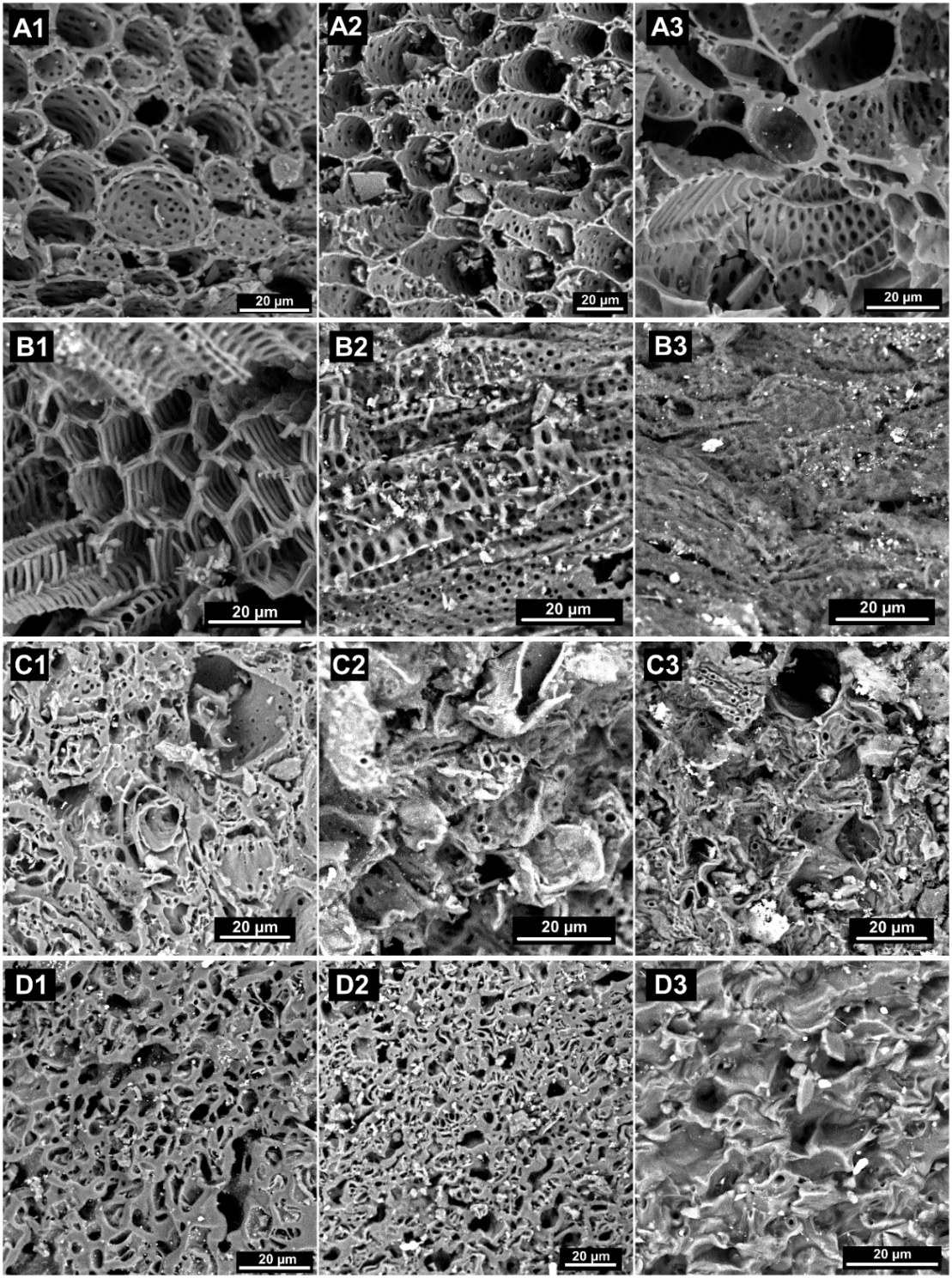


**Figure S6.** SEM pictures of biochar samples before and after CO_2_ activation: corncobs - A1 biochar, A2 0.5 h CO_2_ activation, A3 1 h CO_2_ activation; coconut shells - B1 biochar, B2 0.5 h CO_2_ activation, B3 1 h CO_2_ activation; walnut husks - C1 biochar, C2 0.5 h CO_2_ activation, C3 1 h CO_2_ activation; pistachio husks - D1 biochar, D2 0.5 h CO_2_ activation, D3 1 h CO_2_ activation.

| 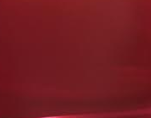 | 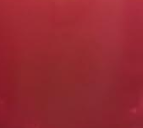 | 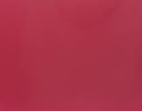 | 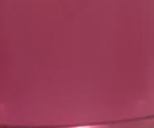 | 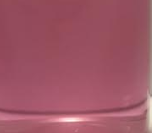 | 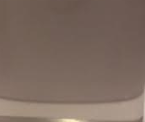 | 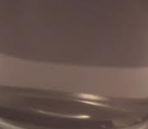 |
| --- | --- | --- | --- | --- | --- | --- |
| 0. BLANK | **1.** 5 mg AC | **2.** 20 mg AC | **3.** 50 mg AC | **4.** 80 mg AC | **5.** 100 mg AC | **6.** 150 mg AC |

**Figure S7.** The solution of Rhodamine B (50 ppm): 0. BLANK - without addition of AC and 1.-6. with addition of various dosages (5-150 mg) of AC (corncob-derived AC, 0.5 h CO2 treatment).

**Table S4**. The ratio between the amounts of Rhodamine B and the adsorption capacity (*q*) and the percent of the adsorbed dye.

| **Before Adsorption** | | | **After Adsorption** | |
| --- | --- | --- | --- | --- |
| **No.** | **AC**  **[mg]** | ***q* [mg g^-1^]** | **RhB**  **[ppm]** | **RhB Removal [%]** |
| 1 | 10.0 | 7.20±0.25 | 49.10±0.43 | 1.81±0.20 |
| 2 | 20.0 | 5.65±0.21 | 27.40±0.36 | 45.23±0.57 |
| 3 | 35.0 | 5.14±0.16 | 14.00±0.34 | 80.07±0.51 |
| 4 | 50.0 | 4.67±0.18 | 3.31±0.10 | 93.41±0.34 |
| 5 | 80.0 | 3.00±0.10 | 2.03±0.09 | 96.02±0.33 |
| 6 | 100.0 | 2.44±0.10 | 1.17±0.09 | 97.86±0.31 |
| 7 | 150.0 | 1.65±0.05 | 0.52±0.06 | 99.04±0.57 |

**Table S5**. The ratio between the amounts of chromium(VI) and the adsorption capacity (*q*) and the percent of the adsorbed dye.

| **Before Adsorption** | | | **After Adsorption** | |
| --- | --- | --- | --- | --- |
| **No.** | **AC**  **[mg]** | ***q* [mg g^-1^]** | **RhB**  **[ppm]** | **RhB Removal [%]** |
| 1 | 5.0 | 10.01±0.31 | 14.92±0.34 | 40.24±0.42 |
| 2 | 7.5 | 8.33±0.27 | 12.34±0.31 | 49.61±0.51 |
| 3 | 10.0 | 6.58±0.18 | 11.91±0.27 | 52.4±0.34 |
| 4 | 12.5 | 5.63±0.23 | 11.09±0.27 | 56.24±0.14 |
| 5 | 15.0 | 5.06±0.19 | 9.71±0.30 | 61.46±0.33 |
| 6 | 17.5 | 4.52±0.20 | 9.26±0.24 | 63.21±0.27 |
| 7 | 20.0 | 4.05±0.15 | 8.84±0.26 | 64.84±0.31 |
| 8 | 25.0 | 3.37±0.11 | 8.15±0.29 | 67.71±0.45 |
| 9 | 30.0 | 2.84±0.09 | 7.90±0.20 | 68.43±0.43 |

a)

b)



**Figure S8**. Specific capacitance (*C*_s_) values calculated from galvanostatic charge and discharge curves recorded at various current densities for corncobs-derived: a) biochar and b) CO_2_-activaled sample (0.5 h of activation); Insets present corresponding charge-discharge curves at 5, 10 and 50 A g^-1^.

1. Pointner, M.; Kuttner, P.; Obrlik, T.; Jäger, A.; Kahr, H. Composition of corncobs as a substrate for fermentation of biofuels. *Agron. Res.* **2014**, *12*, 391–396.

2. Agro-Industrial Wastes as Feedstock for Enzyme Production | ScienceDirect Available online: https://www.sciencedirect.com/book/9780128023921/agro-industrial-wastes-as-feedstock-for-enzyme-production (accessed on Jul 14, 2021).

3. Li, X.; Liu, Y.; Hao, J.; Wang, W. Study of almond shell characteristics. *Int. J. Energy Prod. Manag.* **2018**, *11*, 34–43.

4. Szyszlak-Bargłowicz, J.; Zając, G.; Kuranc, A.; Słowik, T.; Dudziak, A.; Stoma, M.; Wasilewski, J. Chemical properties of selected agri-food industry waste products in the aspect of their use for energetics purposes. *Przem. Chem.* **2018**, *97*, 779–783.
